# Fracture-induced pain-like behaviours in a femoral fracture mouse model

**DOI:** 10.1007/s00198-021-05991-7

**Published:** 2021-06-02

**Authors:** R. Magnusdottir, S. Gohin, F. ter Heegde, M. Hopkinson, I.F. McNally, A. Fisher, N. Upton, A. Billinton, C. Chenu

**Affiliations:** 1 Skeletal Biology Group, Department of Comparative Biomedical Sciences, Royal Veterinary College, 4 Royal College Street, London, NW1 0TU UK; 2 Transpharmation Ltd., The London Bioscience Innovation Centre, 2 Royal College Street, London, NW1 0NH UK; 3 Astrazeneca, Neuroscience, BioPharmaceuticals R&D, AstraZeneca, Cambridge, CB21 6GH UK

**Keywords:** Fractures, Mouse model, Nerve growth factor, Osteoporosis, Pain behaviours

## Abstract

**Summary:**

This study is the first comprehensive characterisation of the pain phenotype after fracture using both evoked and naturalistic behaviours in adult male and ovariectomised female mice. It also shows that an anti-nerve growth factor (NGF) therapy could be considered to reduce pain after fracture surgery.

**Introduction:**

Bone fractures are common due to the ageing population and very painful even after healing. The phenotype of this pain is still poorly understood. We aimed to characterise it in a femoral fracture model in mice.

**Methods:**

We employed both adult male, and female ovariectomised (OVX) mice to mimic osteoporotic fractures. Mice underwent a unilateral femoral fracture maintained by an external fixator or a sham surgery*.* Pain behaviours, including mechanical and thermal sensitivity, weight bearing and LABORAS, were measured from baseline to 6 weeks after fracture. The effect on pain of an antibody against nerve growth factor (anti-NGF) was assessed. Changes in nerve density at the fracture callus were analysed by immunohistochemistry.

**Results:**

Following surgery, all groups exhibited high levels of invoked nociception. Mechanical and thermal hyperalgesia were observed from 1 week after surgery, with nociceptive sensitization in the fracture group maintained for the 6 weeks, whereas it resolved in the sham group after 3 weeks. OVX induced reduction in pain thresholds, which was maintained after fracture. The frequency of naturalistic behaviours did not change between groups. Anti-NGF administered before and weekly after surgery alleviated fracture-induced mechanical nociception. The density of nerve fibres in the fracture callus was similar in all groups 6 weeks after surgery.

**Conclusions:**

Fractures in rodent models are highly painful in both sexes. This pain-like phenotype is prolonged and should be routinely considered in fracture healing studies as it can affect the study outcome. The anti-NGF alleviates fracture-induced mechanical pain.

**Supplementary Information:**

The online version contains supplementary material available at 10.1007/s00198-021-05991-7.

## Introduction

Osteoporosis is the most common bone disease that manifests by bone fractures which represent an international burden, resulting in more cost and disability than any other chronic bone diseases [1].

Substantial pain is one of the most feared complications of bone fractures [2], and there are to date no analgesics that target specifically bone pain which impacts on the quality of life of millions of patients worldwide. The pain is most likely ascribed to nociceptors expressed in the periosteum, which respond to noxious stimuli such as tissue damage induced by fracture [3]. Bone is densely innervated by both sensory and sympathetic nerve fibres [4, 5]. Sensory nerves in bone are mostly thinly myelinated nerve fibres (A-delta) and unmyelinated Calcitonin Gene Related Peptide (CGRP)-positive C-fibres that are highly expressed in the periosteum. Most of them also express tropomyosin receptor kinase A (TrkA), a receptor for nerve growth factor (NGF), a neurotrophin involved in the generation and maintenance of pain states [6]. A novel treatment to reduce fracture-induced pain may be the blockade of NGF/trkA signalling using anti-NGF monoclonal antibodies as they have proven to be effective in reducing pain in several skeletal conditions, including fractures [7–10]. The choice of adequate fracture-associated pain treatment is limited, as some of the currently available analgesics may impair fracture healing [11, 12].

Osteoporosis on its own can be painful [13], and osteoporotic fractures are generally more painful than fractures in healthy bone [14]. Differences in pain and pain inhibition have also been described between men and women, with increased pain sensitivity described in women [15]. However, the characterisation and the mechanisms of pain in osteoporotic fractures have been poorly studied.

With the development of more consistent rodent models of fracture repair, most studies now use these models to assess factors affecting fracture healing. This confronts animal welfare as pain-like behaviour is poorly reported nor controlled in these models [16], despite better understanding and assessment of pain in laboratory animals [17]. Several studies using a rat tibia fracture model of complex regional pain syndrome have, however, reported pain behaviours in this model and shown that anti-NGF antibodies could have strong anti-nociceptive effects [18–20]. Tools to evaluate spontaneous and evoked behaviours which are indicative of pain in rodents have rapidly expanded, allowing the classification and the quantification of the type of pain. Pain severity is determined as the nociceptive response to von Frey hairs [21] and non-noxious cold stimuli, taken respectively as measures for mechanical and thermal allodynia. Static weight-bearing, wherein a reduced weight borne on the ipsilateral hind limb is indicative of increased sensitivity [22], and overnight naturalistic behaviours, with a decrease in overall movement and specific behaviours being indicative of general and ongoing sensitivity [23], are taken as non-evoked sensitivity measurements.

The aim of our study was to quantify pain behaviours during fracture repair in both healthy male and ovariectomy-induced osteoporotic female mice in a common femoral fracture model with an external fixator, using this array of evoked and non-evoked behavioural assays. We also tested the effect of an anti-NGF monoclonal antibody (MEDI578) on these pain behaviours after fracture in female mice and quantified bone innervation in the fracture callus.

## Methods

All animal procedures were performed by trained investigators under PPL 70/8782 in accordance with the Animal (Scientific Procedures) Act, 1986 and the Royal Veterinary Colleges’ Animal Welfare and Ethical Review Body. ARRIVE guidelines were followed for experimental mice and procedures.

### Animals and study design

#### Fracture surgery in male mice

Male mice (C57BL/6, 11–12 weeks old at fracture surgery, Charles River) were acclimatised to the Biological Services Unit (BSU) for 1 week before start of procedures. The animals were kept on a 12-h light/dark cycle with food (LabDiet, PMI Nutrition International, MO, USA) and water ad libitum in groups of one to three on Alpha-dri bedding (Shepherds Speciality Papers, TN, USA). Baseline behaviour measurements were acquired in the week before surgery. Baseline mechanical sensitivity threshold scores were used to randomise the animals to two groups (*n* = 8/group), a group that had unilateral femoral osteotomy (fracture) and a sham surgery group (sham) with the same surgery as the fracture group except that the bones were not drilled into, the fixator was not placed and there was no osteotomy. This allows us to differentiate between surgical pain and bone pain. To ensure comparable behavioural baseline values between groups, high and low responders were spread evenly over different experimental groups. Power analysis of a pilot study data led to a minimum of eight animals in each group for behaviour measurements. At the end of the experiment, the mice were sacrificed with perfusion fixation to preserve the nerves.

#### Ovariectomy of female mice and fracture surgery

Female mice (C57BL/6, 10–11 weeks old, Charles River) were ovariectomised (OVX) or had a sham surgery (sham), as described previously [24], and received postoperative analgesia (buprenorphine) for 2 days after surgery. We chose to study pain behaviours in young mice to solely examine the effect of oestrogen deficiency on pain excluding the effects of ageing which have distinct mechanisms of action. Before ovariectomy, baseline von Frey values were used to randomly assign animals to one of three groups: ovariectomy with a fracture (OVX-fracture, *n* = 12); ovariectomy with a sham fracture (OVX-sham, *n* = 10), and sham ovariectomy with a fracture (sham-fracture, *n* = 6). This was performed as described for male mice. The success of ovariectomy was verified by increased body weight and a loss in trabecular bone at euthanasia. Four weeks after ovariectomy, an osteotomy or a sham fracture surgery was performed as described below. Pain behaviours were measured weekly until 6 weeks after fracture when the animals were euthanized and femurs collected.

#### Pharmacological validation of the fracture pain-like behaviours using an anti-NGF antibody

Non-ovariectomised adult female mice (*N* = 18, C57BL/6, 11–12 weeks old at time of fracture surgery, weight *M* = 21.27 g, range 19.30–24.30 g, Envigo) were used for this study. The mice were allocated to 2 groups (n = 9/group) based on von Frey scores: fracture-MEDI578 (anti-NGF antibody) and fracture-NIP228 (isotype control antibody). MEDI578 and NIP228 were administered subcutaneously before and weekly after fracture surgery at 3 mg/kg. Antibody dilutions were made fresh each week. Before injection, each mouse was weighted for accurate dosing. Pain behaviours were acquired from baseline and measured weekly until 6 weeks after fracture when the animals were euthanized.

#### Bone collection

Ipsilateral femurs were collected 6 weeks after fracture surgery. The animals were terminally anaesthetized (0.25 mL sodium pentobarbital, IP), and the femurs collected after transcardial perfusion using 20 mL ice-cold heparinized 0.01 M phosphate-buffered saline (PBS) (pH 7.4) followed by 20 mL of fixative (12.5% picric acid, 4% paraformaldehyde in 0.01 M PBS, pH 6.9). The animals were dissected on ice and the femurs post-fixed in the same fixative for 48 h at 4 °C. The bones were washed with cold 0.01 M PBS and kept immersed in 0.01 M PBS at 4 °C until micro-CT analysis of bone architecture and immunohistochemistry of nerve fibres were performed.

### Fracture surgery model

Anaesthesia was induced with isoflurane and maintained with isoflurane throughout the surgery and post-surgery x-ray. Buprenorphine (100 μL subcutaneous; 3% Vetergesic, Ceva, UK) was administered before induction of anaesthesia. The fracture surgery was performed as previously described [25, 26]; briefly, a 13-mm incision was made on the right thigh and a blunt dissection performed to visualise the femur. Care was taken to visualise and avoid manipulating the sciatic nerve. A local anaesthetic (0.1 mL mepivacaine hydrochloride, Intra-Epicaine 5 mg/mL, Dechra, UK) was applied to the femur and surrounding muscles. An external fixator made of plastic (MouseExFix Simple L, RISystem, Landquart, Switzerland) was fixed to the anterolateral side of the femur with four titanium pins. The fixator and pins combined weigh 0.20 g (± 0.01 g). A wire saw was passed underneath the femur, and a transverse osteotomy (0.4 mm) performed mid-way between the middle pins through the full thickness of the bone. Postoperative x-rays (HFX90v, 70 kV, 0.8 mAs/s) were acquired of all animals with external fixators whilst under anaesthesia from the surgery, to confirm correct placement of the fixator and pins. Postoperative analgesia was administered twice a day as buprenorphine in jelly, for 3 days.

### Measurement of pain behaviours

Baseline behaviour measurements in male mice were performed before surgery for baseline, resumed 3 days after surgery and continued for 6 weeks post-surgery (days 3, 7, 10, 14, 17, 21, 24, 28, 31, 35, 38 and 42 after surgery). Female mice were tested a week before OVX surgery (week-1), 1 and 3 weeks after OVX and then weekly after fracture surgery. Behavioural analysis conducted included von Frey measurements to measure mechanical sensitivity thresholds, cold plate to measure thermal sensitivity thresholds, weight-bearing to measure asymmetry in stance and Laboratory Animal Behaviour Observation Registration and Analysis System (LABORAS) to quantify naturalistic behaviours. Extended methods for behavioural measurements are found in the supplementary methods. Tests were performed in the following order to prevent iatrogenic effects: von Frey, static weight-bearing, cold plate and LABORAS. LABORAS was performed once a week (in male mice on days 7, 14, 21, 28, 35 and 42; in female mice in weeks 1, 3 and 6) whilst other tests were performed twice weekly in males and once a week in females. All testing was performed by the same experimenter in the daylight phase and at the same time of the day, except LABORAS which was recorded in the night phase over 12 h. Due to the nature of experimental groups and the visibility of the external fixator, the experimenter was unable to be blinded to fracture of sham conditions. The experimenter was blinded to ovariectomised or sham operated mice.

### Micro-computed tomography analysis of trabecular bone in the femur

To confirm the bone loss induced by OVX, trabecular bone in the epiphysis of the femur was compared between sham and OVX female mice using microcomputed tomography (μCT; 5 μm/pixel, 50 kV, 200 μA, Skyscan 1172F, Bruker, Belgium). Projection images of femurs were reconstructed in NRecon (Bruker, Belgium) and datasets reorientated in Dataviewer (Bruker, Belgium). Regions of interests of 200 slices of proximal epiphysial trabecular bone were selected in CTAn and histomorphometric parameters (bone volume (BV), tissue volume (TV) and bone mineral density (BMD)) were analysed in Batman (Bruker, Belgium). Results were exported for statistical analysis, as previously performed [24]. Volume renderings were produced and images exported using CTvox (Bruker, Belgium).

### Immunohistochemistry of nerve fibres

Femurs were decalcified in 10% ethylenediaminetetraacetic acid (EDTA, Sigma-Aldrich) in 0.01 M PBS at 4 °C for 7–10 days until no calcified tissue was visible by μCT. EDTA was changed after 5 days. Upon decalcification, the bones were washed in 0.01 M PBS three times and cryoprotected in 30% sucrose (Sigma-Aldrich) in 0.01 M PBS for at least 48 h and kept in the same media until sectioning. Femurs were placed with the condyles facing down in embedding moulds and immersed in optimal cutting temperature (OCT, Sakura, CA, USA) whilst held in place with tweezers, ensuring correct alignment whilst they were frozen on dry ice. The blocks were then transferred to a cryostat (Bright OTF500, Hacker Industries Inc., USA) and cut at 30 μm in the transverse plane using low profile knives and thawed onto Superfrost Plus slides (Thermo Fisher Scientific, MA, USA). Sections were stained to label nerves, as described previously [27]. The antibodies used were the following: CGRP, a neuropeptide highly expressed by sensory nerves (1:5000, Sigma-Aldrich); tyrosine hydroxylase (TH), a marker for sympathetic nerves (1:1000, Merck Millipore, MA, USA); DAPI (1:20,000, Sigma-Aldrich) for staining of nuclei; and a secondary antibody coupled to fluorescent Cy2 (1:200, Jackson ImmunoResearch, PA, USA).

### Quantification of nerve fibres in the fracture callus and bone marrow

Immunolabelled frozen longitudinal sections were used for nerve analysis, allowing for choice of the same anatomical location in the fracture callus and bone marrow between animals. They were imaged with a Leica SP5 confocal microscope with Leica Application Suite advanced fluorescence software (version 2.6, Leica Microsystems, UK), equipped with a 405-nm diode laser to identify DAPI. Serial confocal Z-stacks of the samples were acquired and nerve length and number analysed visually. For each nerve marker, a confocal Z-stack of images in 1-μm increments was acquired in the fracture callus area (periosteum and bone marrow). In sham animals, images were acquired in a corresponding area to the fracture callus. To account for the quantity of individual nerve fibres in a section, the total number of nerves in each of the two bone regions was counted; where a nerve branched, a new nerve was counted. The image analysis was performed by the same researcher blinded to the mice conditions.

### Statistics

Graphs were produced using GraphPad Prism (7.02, GraphPad, CA, USA) and statistical analysis performed using repeated-measures two-way ANOVA or ordinary one-way ANOVA with Dunnett’s and Tukey’s post hoc tests, and two-sample t tests in Prism. Values are presented as means (*M*) ± *SEM* (standard error of the mean). Values are rounded to two digits. Exact *p* values are given, except where *p* < 0.001, where *p* is presented as *p* < 0.001.

## Results

### Male mice exhibit a clear behavioural phenotype indicative of nociception after fracture

**Fig. 1:**
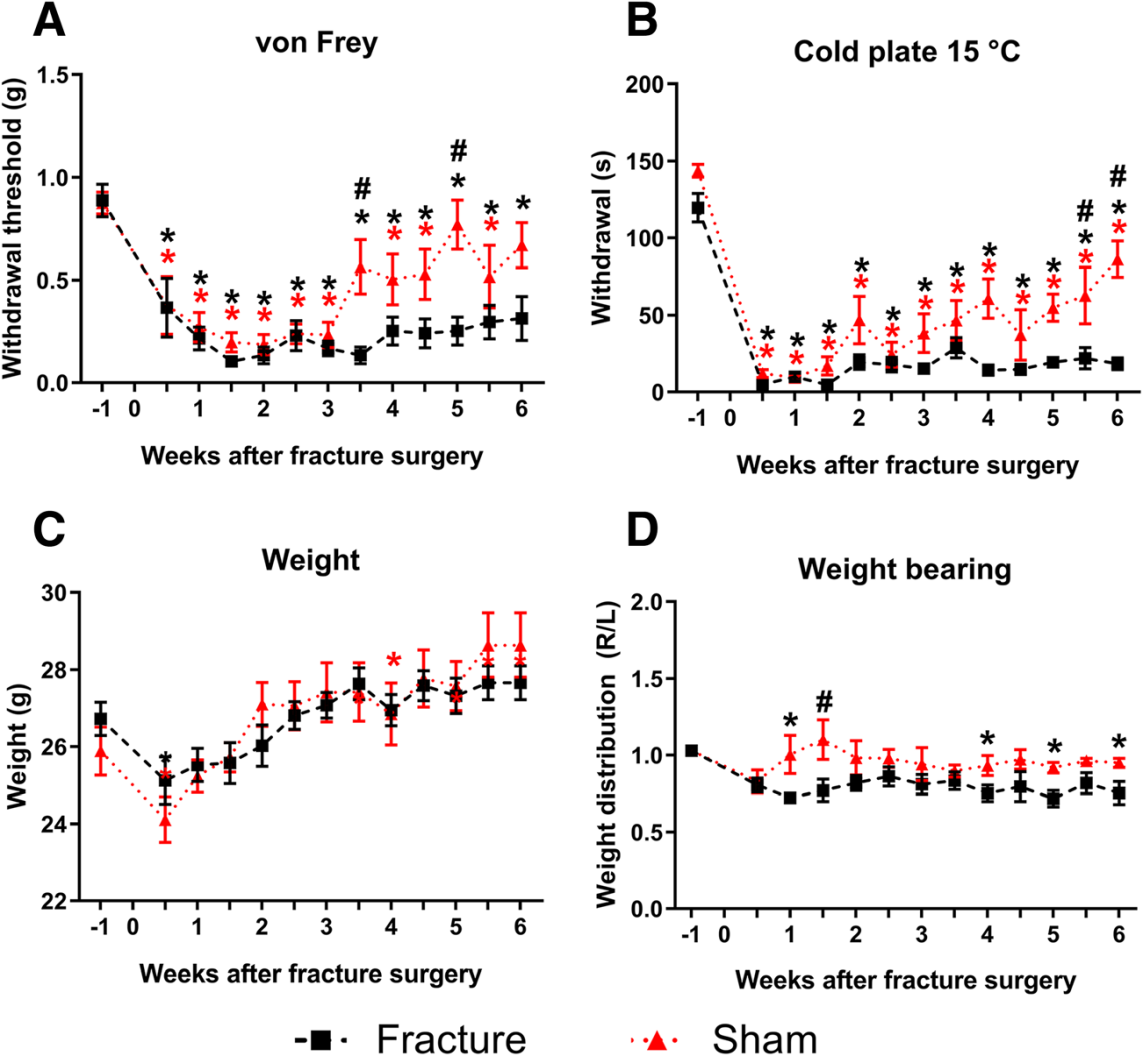
Mechanical and thermal hyperalgesia in male mice in a model of fracture with external fixation. Male C57BL/6 mice, 11–12 weeks old at fracture surgery (week 0). **A** Mechanical hyperalgesia (withdrawal threshold) from baseline (week −1) to 6 weeks after surgery. **B** Thermal hyperalgesia (latency). **C** Weight distribution in the hind legs. **D** Weight. Results are expressed as *M* ± *SEM*, *n* = 8/group. Two-way repeated-measures ANOVA, Dunnett’s multiple-comparisons test for differences within each group: *p < 0.05 each group compared to baseline. Tukey’s multiple-comparisons test for differences between groups; #*p* < 0.05 sham vs fracture

To characterise pain behaviours after fracture in male mice, we used a battery of tests to measure nociception during the fracture healing period. Our results demonstrate that mice exhibit clear pain-like behaviours from 3 days after surgery and through the duration of the 6-week study (Fig. 1A–D). Mechanical hyperalgesia is established in both groups 3 days after surgery, and the sham group differentiates from the fracture group in week three after surgery, with mechanical sensitivity levels returning to baseline values (Fig. 1A). Cold sensitivity is established 3 days after surgery in both groups; however, the sham group exhibits lower levels of sensitivity compared to the fracture group, with significant differences from 5 weeks post-surgery (Fig. 1B). There is a slight weight loss following surgery, whilst accounting for the added weight of the fixators in the fracture group; however, both groups gain weight throughout the study (Fig. 1C). Weight-bearing is disrupted after surgery in the fracture group, which places less weight on the affected leg than the sham animals do; however weight-bearing returns to baseline values 2 weeks after surgery (Fig. 1D).

**Fig. 2:**
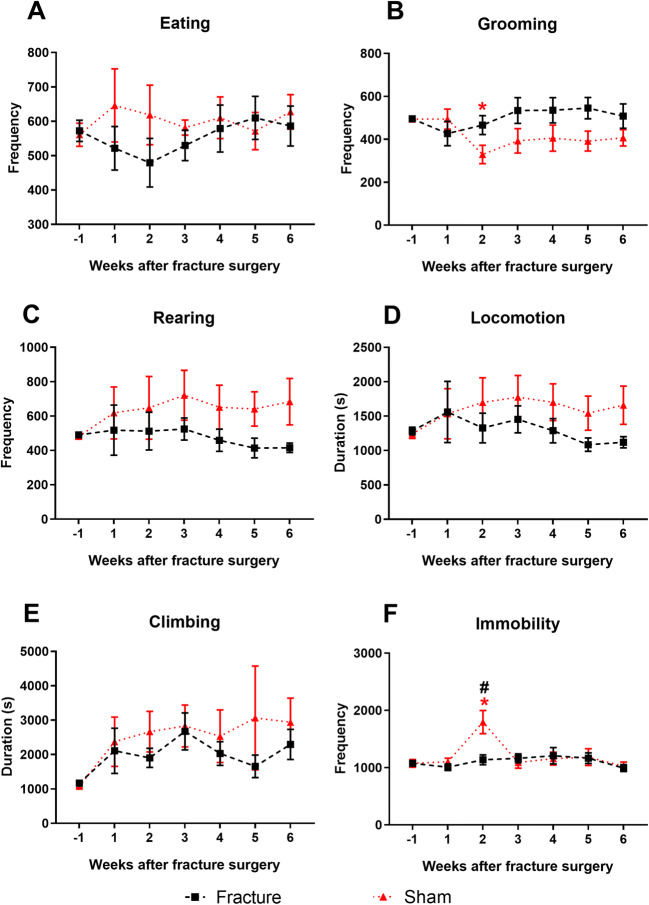
Naturalistic behaviours in male mice in a model of fracture with external fixation*.* Male C57BL/6 mice, 11–12 weeks old at fracture surgery (week 0). Data points represent cumulative behaviour over the night period. **A** Eating frequency from baseline (week − 1) to 6 weeks after surgery. **B** Grooming frequency. **C** Rearing frequency. **D** Locomotion duration. **E** Climbing duration. **F** Immobility frequency. Results are expressed as *M* ± *SEM*, *n* = 8/group. Two-way repeated-measures ANOVA, Dunnett’s multiple-comparisons test for differences within each group: *p < 0.05 each group compared to baseline. Tukey’s multiple-comparisons test for differences between groups; #*p* < 0.05 sham vs fracture

Naturalistic pain behaviours were measured during the night phase weekly from baseline (− 1) to 6 weeks after fracture surgery (Fig. 2A–F). There are no differences in eating behaviours (Fig. 2A); however, grooming is significantly decreased in the sham group 2 weeks after surgery and maintained throughout the study (Fig. 2B). The frequency of rearing (Fig. 2C) and durations of locomotion (Fig. 2D) and climbing (Fig. 2E) are all unaffected by the surgery, although there is a trend for higher locomotion and climbing in the sham group. Immobility is significantly increased in the sham group 2 weeks after surgery, but this is not maintained with time (Fig. 2F).

### Ovariectomy induces changes in nociception

The success of ovariectomy was confirmed by the significant decreases in trabecular bone volume (BV/TV) and bone mineral density (BMD) in the epiphysis of the femur in both OVX groups compared to sham (BV/TV; sham fracture, 10.36%; OVX fracture, 3.89% P < 0.0001; OVX sham, 6.52%; P < 0.01; BMD; sham fracture, 0.21 g/cm^3^; OVX fracture, 0.12 g/cm^3^ P < 0.001; OVX sham, 0.15 g/cm^3^ P < 0.01).

**Fig. 3:**
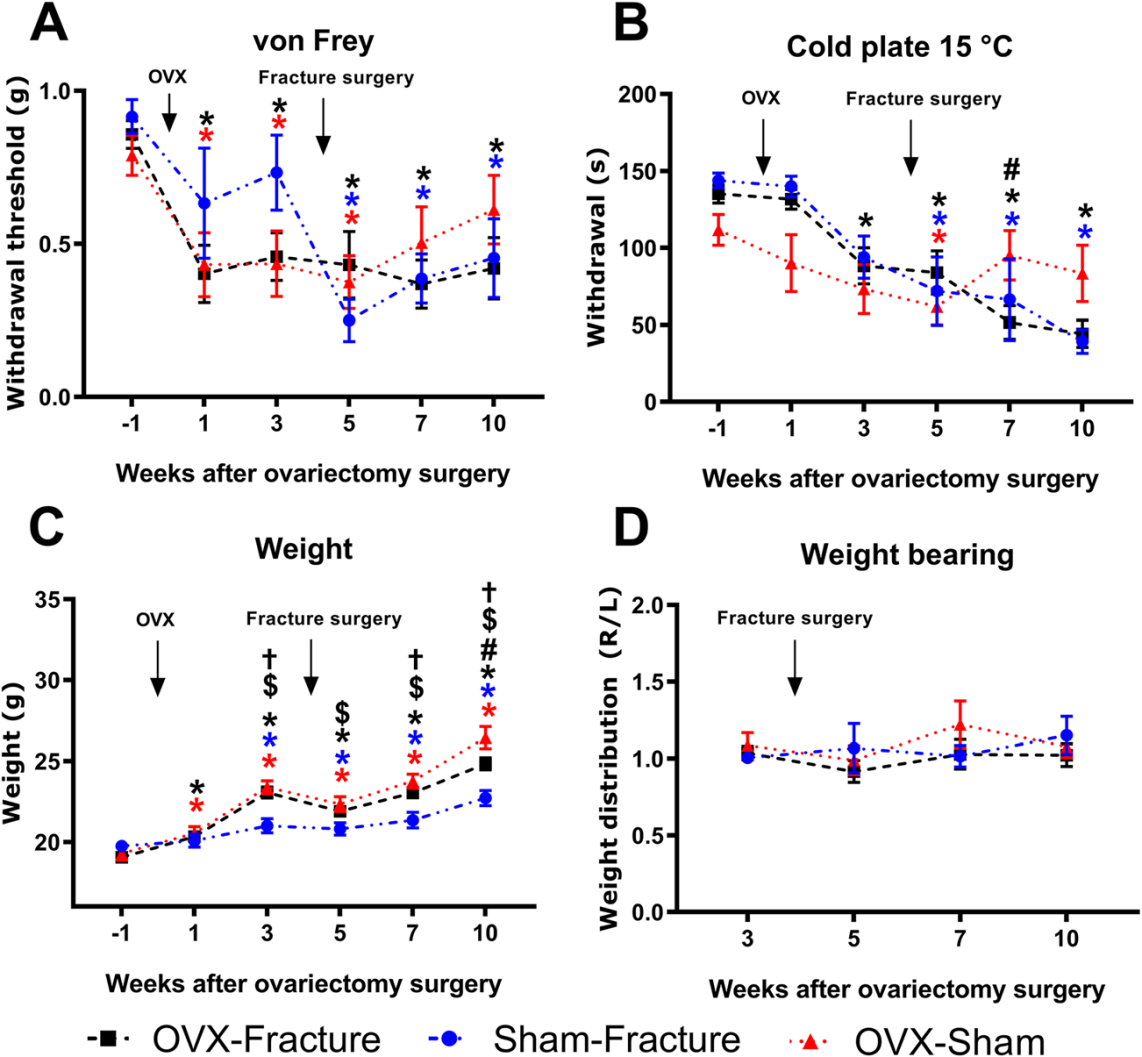
Mechanical and thermal hyperalgesia in ovariectomised female mice in a model of fracture with an external fixation. Female C57BL/6 mice were ovariectomised at 10–11 weeks old (week 0) and fracture surgery performed 4 weeks after ovariectomy (OVX) (week 4). We had three groups, ovariectomy with fracture (OVX-fracture), ovariectomy with a sham fracture surgery (OVX-sham) and a sham ovariectomy with fracture (sham-fracture). **A** Mechanical hyperalgesia (withdrawal threshold) from baseline (week − 1) to 10 weeks after ovariectomy surgery. **B** Thermal hyperalgesia (latency). **C** Weight. **D** Weight distribution from 1 week before fracture surgery. Results are expressed as *M* ± *SEM*, sham-fracture; *n* = 6, OVX-sham; *n* = 10, OVX-fracture; *n* = 12. Two-way repeated-measures ANOVA, Dunnett’s multiple-comparisons test for differences within each group: *p < 0.05 each group compared to baseline. †*p* < sham-fracture compared to OVX-fracture, $*p* < 0.05 sham-fracture compared to OVX-sham, #*p* < 0.05 OVX-sham compared to OVX-fracture

To characterise the OVX-induced pain-like behaviours in female mice, pain scorings on von Frey and cold plate were measured before OVX (baseline, week −1) and 1 and 3 weeks after OVX (Fig. 3A–D). Ovariectomy induces a significant reduction in the mechanical withdrawal threshold in the OVX-fracture and OVX-sham groups compared to baseline (Fig. 3A). The thermal sensitivity is also significantly reduced in the OVX-fracture group 3 weeks after ovariectomy (Fig. 3B).

### Female OVX mice exhibit a clear behavioural phenotype indicative of nociception after fracture

The addition of a fracture after the animals had been ovariectomised for 4 weeks did not increase the mechanical hyperalgesia observed after OVX (Fig. 3A). In contrast, there was a sharp increase in mechanical sensitivity scores in the sham-fracture group after fracture which is significantly different from the baseline (Fig. 3A).

The OVX-sham group differentiates from the two fracture groups 3 weeks after fracture (7 weeks after OVX) where their scores reduce in severity (Fig. 3B). Six weeks after fracture surgery, both fracture groups (OVX-fracture and sham-fracture) have significant thermal hyperalgesia whilst the OVX-sham group has returned to baseline values. As expected, significant weight gain was observed in both ovariectomy groups from 1 week after OVX surgery (Fig. 3D) which continued to increase over the sham OVX group throughout the study. Weight-bearing was measured from before fracture surgery (week 3) to 6 weeks after surgery (week 10) and did not show differences between the groups (Fig. 3D).

**Fig. 4:**
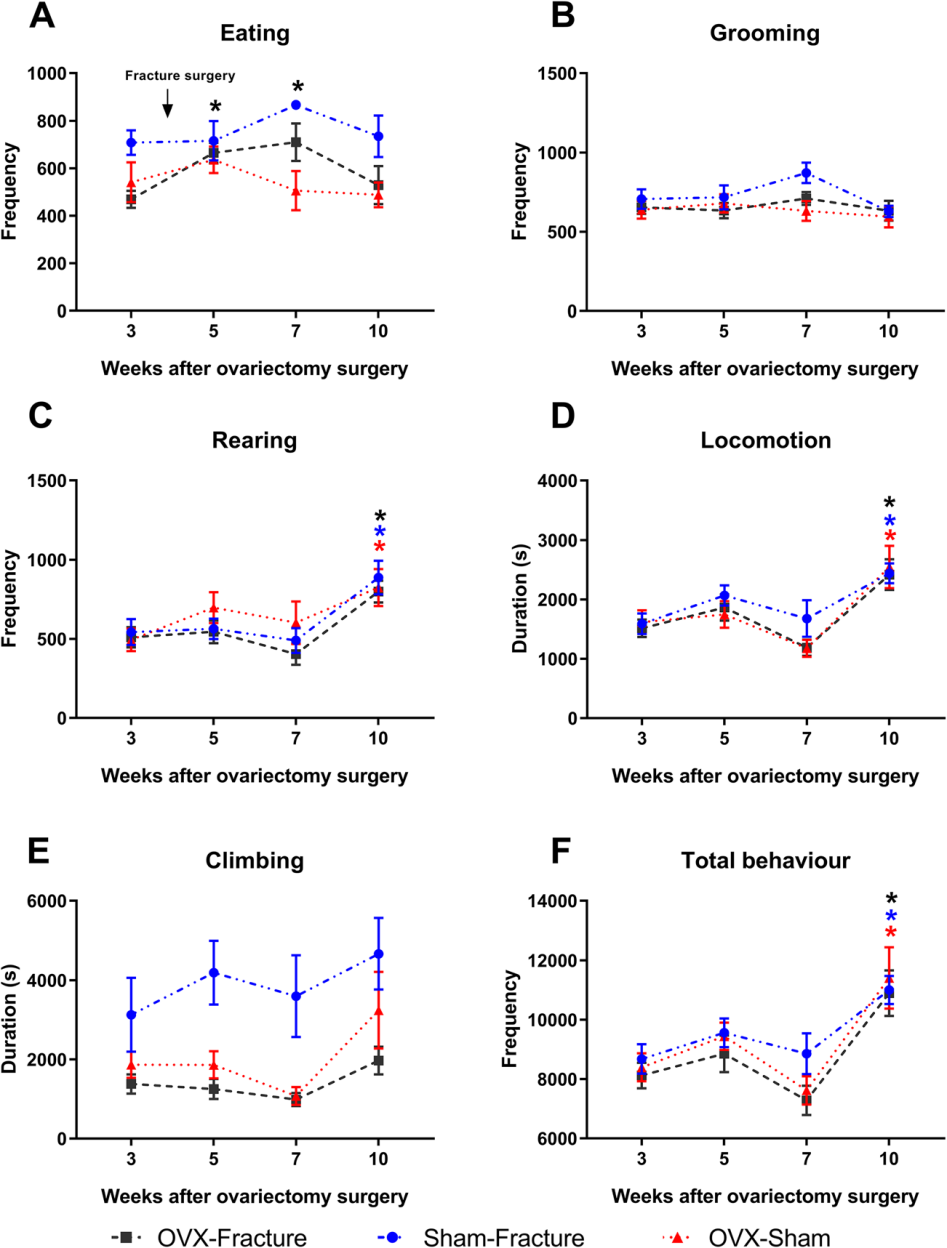
Naturalistic behaviours in ovariectomised female mice in a model of fracture with an external fixation. Female C57BL/6 mice, 14–15 weeks old at fracture surgery (week 4 after OVX). Data points represent cumulative behaviour over the night period of 12 h. We had three groups, ovariectomy and fracture (OVX-fracture), ovariectomy and sham fracture surgery (OVX-sham) and sham ovariectomy with fracture (sham-fracture). **A** Eating frequency from baseline before fracture (week 3 after OVX) to 6 weeks after fracture surgery (week 10). **B** Grooming frequency. **C** Rearing frequency. **D** Locomotion duration. **E** Climbing duration. **F** Immobility frequency. Results are expressed as *M* ± *SEM*, sham-fracture; *n* = 6, OVX-sham; *n* = 8, OVX-fracture; *n* = 8. Two-way repeated-measures ANOVA, Dunnett’s multiple-comparisons test for differences within each group: *p < 0.05 each group compared to baseline. Tukey’s multiple-comparisons test for differences between groups; not significant

Naturalistic behaviours (Fig. 4A–F) were measured in female mice from 1 week before fracture surgery (week 3) until the end of the study. The frequency of eating is significantly increased in weeks 5 and 7 in the sham-fracture group compared to baseline (Fig. 4A). Grooming behaviours are unchanged in all groups (Fig. 4B). Rearing is significantly increased in week 10 in all groups compared to baseline (Fig. 4C) and so is the duration of locomotion (Fig. 4D). Climbing behaviours are unaffected (Fig. 4E). The frequency of all behaviours is significantly increased in week 10 in all groups compared to baseline, but there were no differences between the groups (Fig. 4F).

### The density of innervation in the fracture callus is unchanged by ovariectomy and fracture

**Fig. 5:**
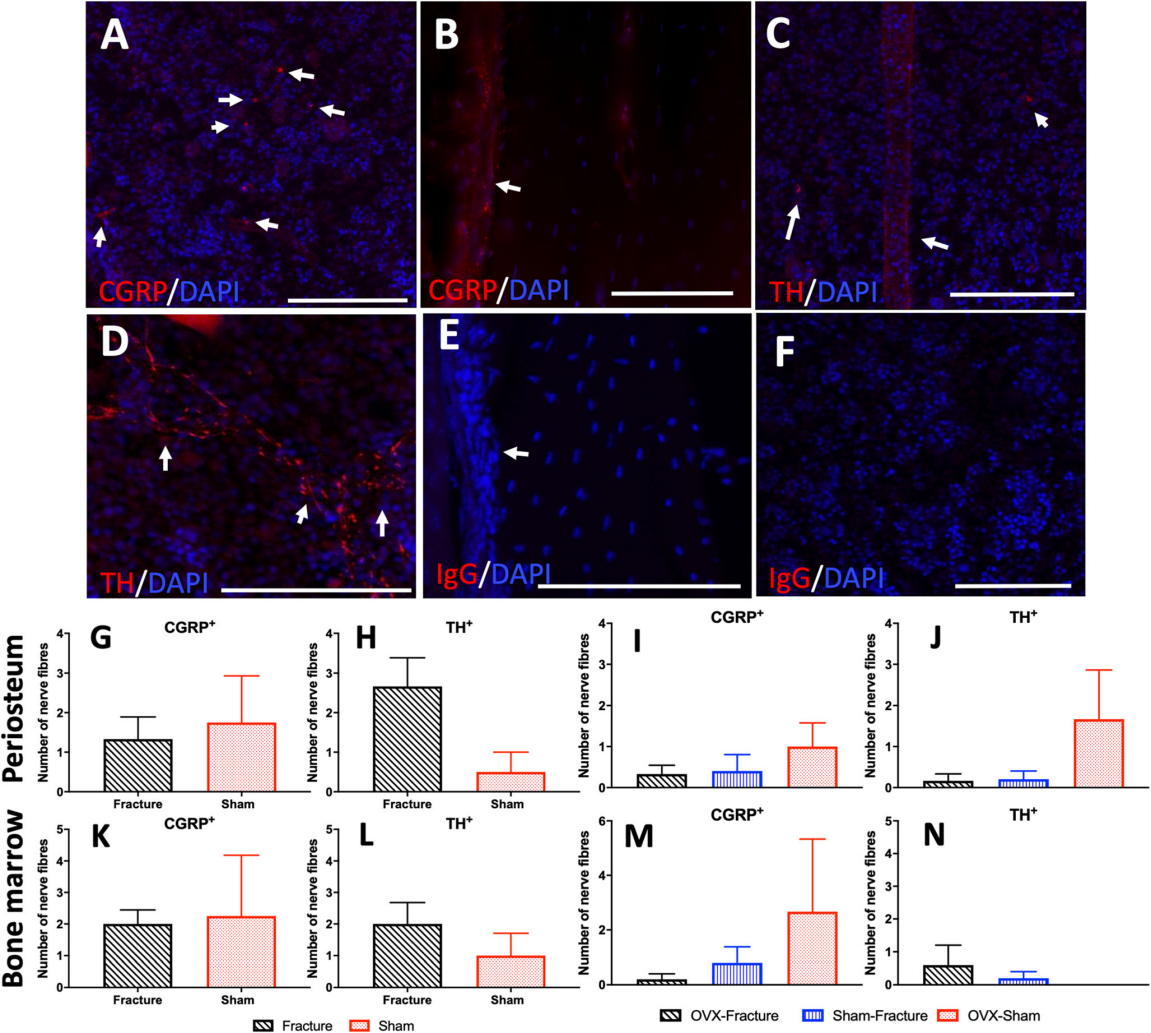
Localization and quantification of sensory (CGRP^+^) and sympathetic (TH^+^) nerve fibres in the femoral fracture callus of male and female mice 6 weeks after fracture surgery. Frozen sections are 30 μm thick in a Z-Stack of confocal images acquired at the fracture callus or a corresponding area in the sham group. They were counterstained with DAPI to label nuclei. **A** CGRP^+^ nerve fibres (arrows) scattered in the bone marrow. **B** A CGRP^+^ nerve fibre (arrow) in the periosteum at the fracture callus. **C** A single TH^+^ nerve in the bone marrow and other smaller TH^+^ nerve fibres (arrows). **D** TH^+^ nerve fibres in the fracture callus (arrows). **E** Frozen section stained with IgG control showing the periosteum at the fracture callus (arrow). **F** Frozen section stained with IgG control showing the bone marrow. **G** Number of CGRP^+^ nerves in the periosteum in male mice. **H** Number of TH^+^ nerves in the periosteum in male mice. **I** Number of CGRP^+^ nerves in the periosteum in female mice. **J** Number of TH^+^ nerves in the periosteum in female mice. **K** Number of CGRP^+^ nerves in the bone marrow in male mice. **L** Number of TH^+^ nerves in the bone marrow in male mice. **M** Number of CGRP^+^ nerves in the bone marrow in female mice. **N** Number of TH^+^ nerves in the bone marrow in female mice. Scale bars 50 μm. Results are expressed as *M* ± *SEM*, fracture *n* = 6, sham *n* = 4. Male mice: two-sample t test: not significant. Female mice: sham-fracture, *n* = 6; OVX-sham, *n* = 5; OVX-fracture, *n* = 4. Ordinary one-way ANOVA: not significant

The number of sensory and sympathetic nerve fibres was analysed in two specific areas at the fracture callus level, the periosteum and bone marrow, both in male and female mice 6 weeks after fracture. Figure 5 panels A and B show the innervation of CGRP+ nerve fibres in the bone marrow (A) and in the fracture callus at the periosteum (B) in male mice whilst panels C and D show the innervation of TH+ nerve fibres in the fracture callus (C) and in the bone marrow (D) in male mice. Figure 5 panels E and F demonstrate the lack of immunostaining in the periosteum (E) and in the bone marrow in male mice with an IgG control antibody.

Despite an increase in TH-positive nerve fibres in the fracture callus, there were no significant differences in the number of CGRP^+^ or TH^+^ nerves between the fracture and sham groups in male mice (Fig. 5G, H, K, L) or in female mice that had been ovariectomised (Fig. 5I, J, M, N). There was also no significant effect of ovariectomy on the innervation of the fracture callus (Fig. 5I, J, M, N).

### Mice treated with the anti-NGF antibody exhibit significantly less mechanical hyperalgesia after fracture than those treated with the control antibody

**Fig. 6:**
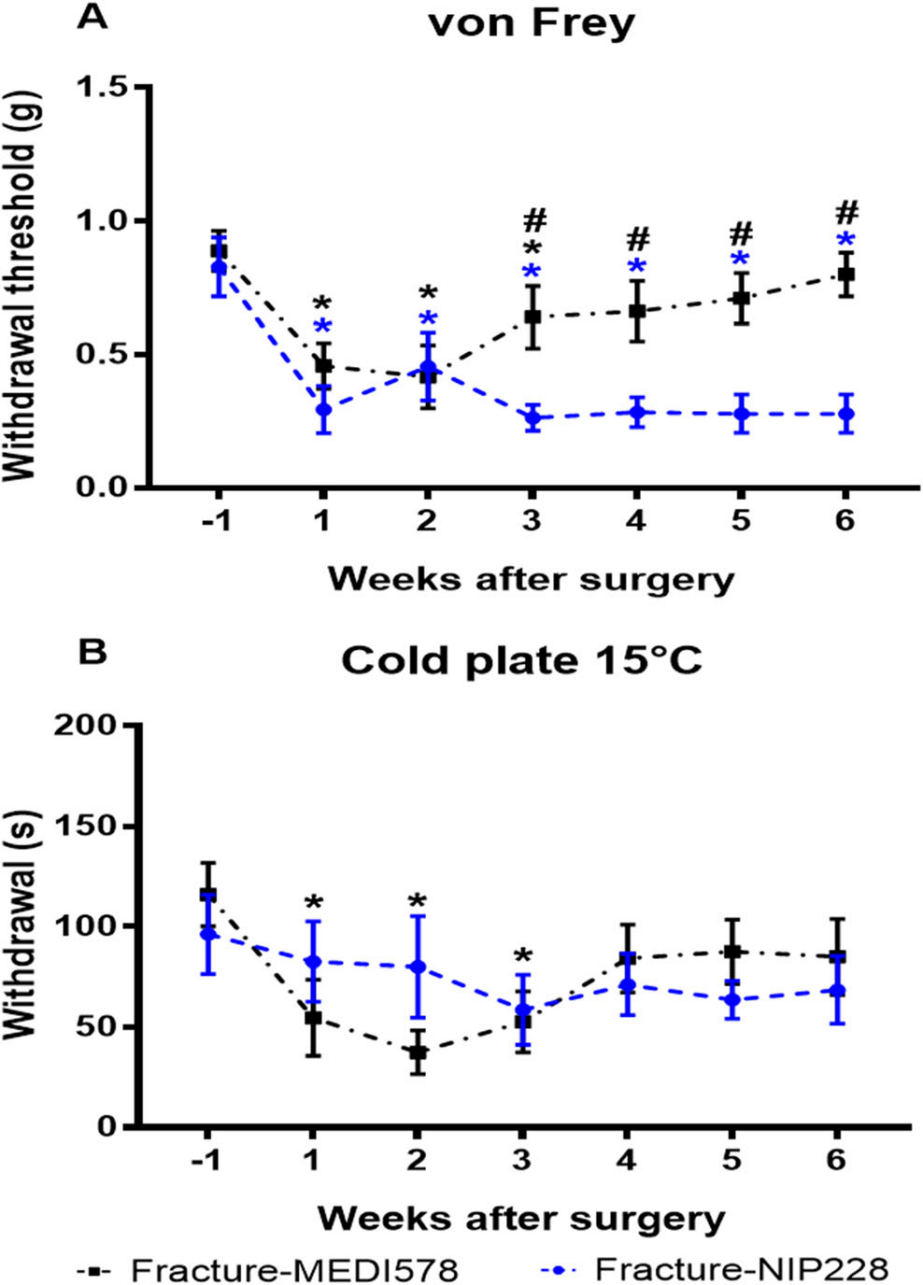
Mechanical and thermal hyperalgesia in female mice treated with the anti-NGF antibody (MEDI578) or control antibody (NIP228) in a model of fracture with external fixation. Female C57BL/6 mice, 11–12 weeks old at fracture surgery (week 0). **A** Mechanical hyperalgesia (withdrawal threshold) from baseline (week −1) to 6 weeks after surgery. **B** Thermal hyperalgesia (withdrawal latency). Results are expressed as *M* ± *SEM*, fracture-MEDI578 *n* = 9, fracture-NIP228 *n* = 9. Two-way repeated-measures ANOVA, Dunnett’s multiple-comparisons test for differences within each group: *p < 0.05 each group compared to baseline. Tukey’s multiple-comparisons test for differences between groups: #*p* < 0.05 fracture-MEDI578 compared to fracture-NIP228

To further validate the pain behaviours observed after fracture in our animal model, we tested the effect of an anti-NGF monoclonal antibody (MEDI578) on evoked behaviours after fracture in healthy female mice compared to a control antibody (NIP 228). Both groups had established significant mechanical hyperalgesia in week 1 compared to baseline (fracture-MEDI578, *p* = 0.0001; fracture-NIP228, *p* = 0.0001) which was maintained in week 2 (fracture-MEDI578, *p* = 0.0001; fracture-NIP228, *p* = 0.0001) *F*(6, 84) = 10.73, *p* < 0.0001 (Fig. 6A). By week 3, the fracture-MEDI578 group still had significant mechanical hyperalgesia compared to baseline (*p* = 0.0356), and there was a significant difference between the groups (*p* = 0.0461) with the fracture-MEDI578 group having lower scores *F*(6, 84) = 4.70, *p* = 0.0004. The mechanical hyperalgesia in the fracture-MEDI578 group resolved by week 4 (*p* = 0.0661). The fracture-NIP228 group continued to have persistent mechanical hyperalgesia throughout the 6-week study, whilst a gradual reduction in the withdrawal threshold was observed in the fracture-MEDI578 group. The difference between the groups was maintained throughout the study. Thermal hyperalgesia was established in the fracture-MEDI578 group in week 1 after surgery (*p* = 0.0130) compared to baseline and was maintained in weeks 2 (*p* = 0.0008) and 3 (*p* = 0.0096) *F*(6, 84) = 2.45, *p* = 0.0287 (Fig. 6B). No changes in the withdrawal latency were observed in the fracture-NIP228 group. There were no differences between the groups (*p* = 0.1829). Naturalistic behaviours showed no significant differences between the groups (data not shown), although the group treated with MEDI578 tended to have increased overall activity. All our fractured mice had unions, and we showed no deleterious effect of the anti-NGF antibody on fracture healing as assessed by micro-CT, 6 weeks after surgery (data not shown).

## Discussion

In the developed world, fractures are the most frequently occurring skeletal disorder and, as such, represent a significant social-economic burden [28]. Complications during fracture healing are often painful and typically arise in patients with comorbidities such as osteoporosis or diabetes [29, 30]. Whilst animal fracture models have been essential for our understanding of bone repair, much remains unknown about the development of pain during fracture healing or whether bone diseases, such as osteoporosis, can influence the pain phenotype.

In this study, we utilised an established model of femoral fracture with external fixation [25, 26] to characterise the extent and nature of pain-like behaviour following fracture. This model has been extensively used to unravel mechanisms responsible for bone regeneration and to investigate ways to improve fracture healing [31]. Although this model of diaphyseal fractures with external fixation does not represent the best pre-clinical model of osteoporosis, it provides increased stability, a requirement for fracture healing, and is highly standardised which has implications for the NC3Rs as smaller groups of mice can be used. Models of metaphyseal and vertebral fractures are difficult to perform in rodents and lead to fracture instability and pain. We show that mice undergoing this fracture surgery with a fracture gap of only 0.4 mm exhibit a sharp drop in behavioural threshold levels, indicative of nociception, immediately after surgery and persisting for up to 6 weeks post-surgery. Although anaesthesia and post-operative analgesia may confound pain behaviour results a few days after surgery, this was not an issue in our studies that lasted for 6 weeks.

This increased hypersensitivity was seen in both mechanical and thermal measurements whilst only minimal changes in naturalistic behaviours were observed. Interestingly, the immediate onset of the pain-like phenotype was also present in the sham mice which only had blunt dissection of the muscles without any fracture. The pain-like phenotype in this group persisted for 3 weeks after which the animals slowly recovered. This post-surgical increased sensitivity was previously observed in different types of sham operations [32] and is likely the result of local inflammation processes which encourage soft tissue healing [33]. In contrast, the fracture group showed persistent mechanical and thermal hypersensitivities for the duration of the study, indicative of a chronic pain-like phenotype.

Even with a wide range of variation in fracture methodology, the nociceptive sensitization seen in this model of fracture with external fixation is comparable to that seen in other models of fracture which typically rely on the insertion of a stainless steel pin into the intramedullary canal of either the tibia or the femur [34–36] to fixate the osteotomy during healing. In these models, ipsilateral mechanical hypersensitivity develops as soon as 3 days post-surgery and lasts up to 4 weeks [35]. This is accompanied by an increase in cold allodynia [34] and reduction in voluntary movement [36]. We show a similar acute onset of mechanical hypersensitivity and cold allodynia but less impact on naturalistic behaviours and spontaneous movement in our fracture model with external fixator. It is possible that the lack of both axial and rotational stability in the intramedullary pin models reduces the mobility of mice following fracture [37]. In contrast, external fixation offers a higher degree of stability, reducing the likelihood of dislocation or other complications, which allows for increased weight-bearing and usage, therefore lessening the impact on naturalistic behaviours.

Despite skeletal pain from osteoporotic fractures being a major health concern in the ageing populations, no preclinical work was done to characterise pain-like behaviour following fracture in osteoporosis animal models. In this study, we used ovariectomised mice as a model for osteoporosis. Whilst the main source of pain in osteoporotic patients is typically considered to be complex or micro fractures due to the lower bone mineral density [13, 14], a subset of these patients have pain without showing signs of fracture suggesting that osteoporotic bone loss itself can be painful [38, 39]. This is supported by numerous reports of mice developing mechanical and thermal hypersensitivity following OVX surgery [40, 41]. Here, we show a similar pain-like phenotype, with OVX mice developing mechanical and thermal hypersensitivity 1 week post-surgery whilst naturalistic behaviour remained unchanged. Interestingly, there was no additional nociceptive sensitization in OVX mice following fracture surgery, suggesting that complex osteoporotic fractures are not more painful than fractures in healthy bone. This is unexpected as oestrogen deficiency has been shown to influence inflammatory processes following fracture, thus causing a decreased fracture healing [42]. Possibly, this decreased fracture healing does not add on to the hypersensitivity that has already developed due to the OVX-induced oestrogen deficiency. It is, however, important to note that although OVX is a widely used model of bone loss, the decrease in oestrogen levels in this model does not necessarily reflect the clinical situation as ovaries produce other hormones and growth factors that may have direct or indirect effects on bone. Further work is needed to compare sex-related differences in fracture-induced pain-like behaviours in a fully randomised study design and to examine the potential role of oestrogens on these behaviours.

Our results demonstrate that the anti-NGF was successful at alleviating fracture-induced mechanical nociception compared to the control antibody. This is in agreement with a previous study describing about 50% reduction in skeletal pain-like behaviour after fracture with the administration of an anti-NGF [10]. The anti-NGF was administered for the first time at the time of fracture surgery in accordance with the literature that describes successful analgesia only when anti-NGF is administered before or simultaneously with the painful stimuli [7, 8]. Various anti-NGF monoclonal antibodies have successfully been used to treat several skeletal-induced pain conditions [7–10]. Our results not only validate our measurements of evoked behavioural assays to evaluate nociception but support the use of an anti-NGF to reduce fracture pain without affecting bone repair, as previously suggested [43].

Additionally, we show in this femoral fracture model that bone innervation is not altered by fracture healing in either healthy or osteoporotic bone. Analysis showed no change in CGRP+ or TH+ nerve volume in either bone marrow or periosteum at the fracture callus or in the mid-diaphysis following OVX surgeries, 6 weeks post-fracture. There was an increased variability in the volume of CGRP+ nerve fibres, with a higher volume in the periosteum in female mice following fracture in both healthy and osteoporotic mice. This contrasts to work done with the intramedullary pin femoral fracture model where it was shown that in complex, non-healed fractures there is an increase in both sensory and sympathetic innervation of the bone marrow and periosteum [44]. This could be explained by the reduced invasiveness of the external fixator model compared to the intramedullary pin fracture model as the combination of unhealed, complex fractures with intramedullary damage could justify the severe increase in innervation seen by Chartier and colleagues [44]. It should be noted that, apart from a local increase in innervation, there are other neuronal changes that can contribute to the development of nociceptive behaviour, including central sensitization or the recruitment of silent nociceptors, and these are typically seen at the level of the dorsal root ganglia or spinal cord [45]. In addition, innervation of the fracture callus is a quick process and it is possible that the time point examined here, 6 weeks after fracture, might be too late in the fracture healing process to see any differences in innervation.

Our study has several limitations, the major one being its translational impact. We used a mouse model to quantify behavioural changes after fractures as measurements for referred pain. These pain behaviours may not necessarily reflect the pain symptoms (hyperalgesia and allodynia) experienced by humans. We show nevertheless that some of these pain behaviours can be reversed by an anti-NGF, suggesting that we can use our model to study fracture pain. Although the anti-NGF was effective in reducing pain-like behaviours after fractures in healthy mice, its effect should be tested in more complex osteoporotic fractures. We also studied pain behaviours in young ovariectomised mice to solely examine the effect of oestrogen deficiency and not ageing. This may be a weakness of our study as young ovariectomised mice may not represent the best preclinical model to reflect the pain experienced by ageing osteoporotic women. Ageing bone is weaker than in young adults, has different material properties and is less vascularised, making it slower to repair. Finally, we only studied pain behaviours for 6 weeks after fracture, which may not be long enough to study long-term pain associated with central sensitization.

With the pandemic of fragility fractures, it is essential to better manage the severe pain associated with those fractures. For this, one needs a better characterisation of this pain during the whole healing process. This study is the first comprehensive characterisation of the pain-like phenotype after fracture using both evoked and naturalistic behaviours in adult male and ovariectomised female mice. Overall, we have demonstrated that fracture surgeries in rodents are painful in both sexes and that an anti-NGF can partly alleviate this pain-like phenotype, suggesting that it could be used as a therapy to manage pain after fracture. Whilst we saw no changes in innervation between groups, it is possible that the prolonged pain-like behaviours after fractures are partly due to nerve sprouting in the bone early in the healing process and that these changes in innervation are resolved by 6 weeks. This pain-like phenotype seen in both fracture and sham animals is an important finding and should be routinely considered in bone regeneration or fracture healing studies.

## Supplementary Information

ESM 1 (DOCX 105 kb).

ESM 2 (DOCX 13 kb).

## Data Availability

The data are stored in the Royal Veterinary College drives and in external hard drives.
