# Supplementary material for: Fracture-induced pain-like behaviours in a femoral fracture mouse model
Source: Osteoporos Int. 2021 Jun 2;32(11):2347–59. doi: 10.1007/s00198-021-05991-7 (PMC8563675; doi:10.1007/s00198-021-05991-7)
Supplement: Supplementary file 1 — (DOCX 105 kb). [file 198_2021_5991_MOESM1_ESM.docx]

**Supplementary Figures**

Figure 1S: Mechanical hyperalgesia is more pronounced in NIP228-treated Sham mice than MEDI578-treated mice after sham surgery while thermal sensitivity remains unchanged.

Female C57BL/6 mice, 11 - 12 weeks-old at fracture Sham surgery (week 0). (A) Mechanical hyperalgesia (withdrawal threshold) from baseline (week -1) to six weeks after surgery. (B) Thermal hyperalgesia (withdrawal latency). Results are expressed as *M* ± *SEM*, Sham-MEDI578 *n* = 10, Sham-NIP228 *n* = 10. Two-way repeated measures ANOVA, Dunnett’s multiple comparisons test for differences within each group: * *p* < 0.05 each group compared to baseline. Tukey’s multiple comparisons test for differences between groups: ns.


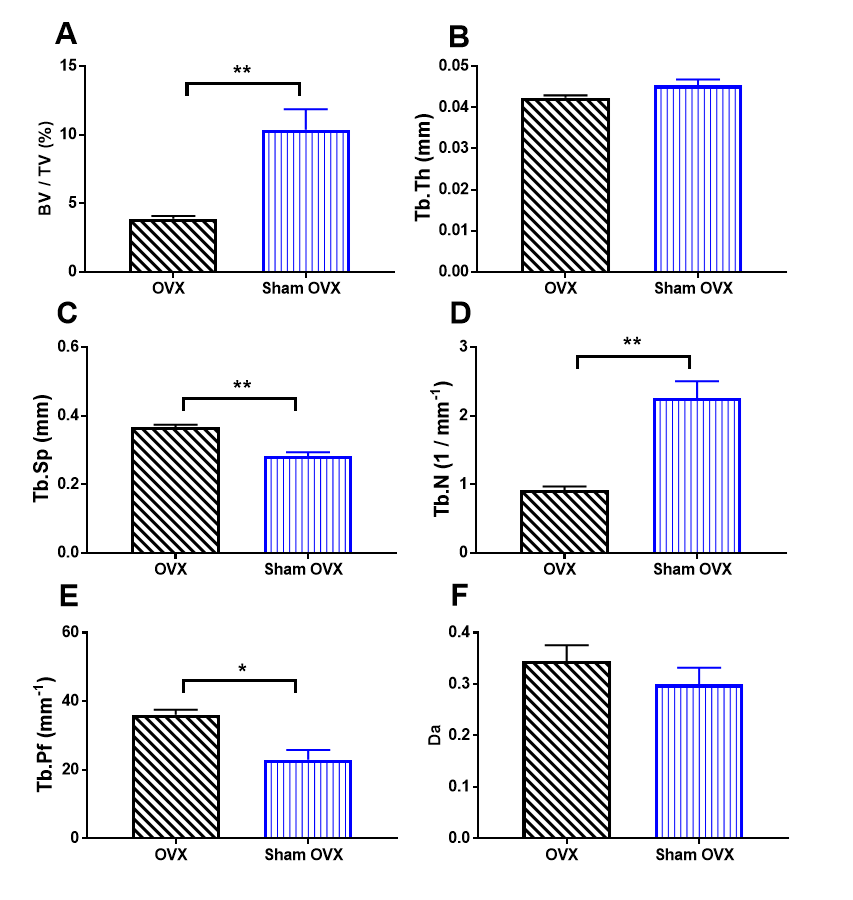


**Figure 2S: Three-dimensional values of the microstructure of trabecular bone in the epiphysis 10 weeks after ovariectomy surgery (six weeks after fracture). These parameters were measured in the femoral epiphysis of the OVX-fracture group (OVX) and the sham-fracture group (sham OVX).**

(A) Bone volume fraction. (B) Trabecular thickness. (C) Trabecular separation. (D) Trabecular number. (E) Trabecular pattern factor. (F) The degree of anisotropy. Results are expressed as *M* ± *SEM*, *n* = 6 / group. BV / TV (%): bone volume fraction, Tb.Th (mm): mean thickness of the trabeculae, Tb.Sp (mm): mean distance between trabeculae, Tb.N (1 / mm^-1^): average number of trabeculae, Tb.Pf (mm^-1^): inverse index of connectivity, DA: the degree of anisotropy. Paired two-tailed *t*-test for differences between groups: * *p* < 0.05, ** *p* < 0.01.
