# Supplementary material for: Fracture-induced pain-like behaviours in a femoral fracture mouse model
Source: Osteoporos Int. 2021 Jun 2;32(11):2347–59. doi: 10.1007/s00198-021-05991-7 (PMC8563675; doi:10.1007/s00198-021-05991-7)
Supplement: Supplementary file 2 — (DOCX 13 kb). [file 198_2021_5991_MOESM2_ESM.docx]

### **Supplementary methods**

### Mechanical allodynia

Mechanical allodynia was monitored with graduated levels (0.07, 0.16, 0.4, 0.6, and 1.0 g) of von Frey filaments (Aesthesio Precision Tactile Sensory Evaluator, DanMic, CA, USA) applied to the right (ipsilateral) hind paw for two seconds, 10 times at each weight (1). The level of response was calculated as the first level to have 5 out of 10 responses positive. A response was classified as a brisk withdrawal of the paw following application, vocalization, or licking of the paw immediately following application.

### Thermal allodynia

The mice were placed on a cold plate (Hot / Cold plate, Bioseb, France) set to 15 °C (± 0.1 °C) and the time to first nociceptive response was monitored. Nociceptive response was classified as jumping, licking of a hind paw, vocalization or a brisk retraction of the hind paw. The maximum time allowed for the test was 150 seconds to prevent tissue damage. Animals were habituated in the test chamber for five minutes twice, before two baseline readings were acquired (average is presented as baseline).

### Static weight-bearing

Animals were placed in a static weight-bearing device (Incapitance Tester, Linton, UK) and positioned with both hind legs on the floor sensors and front legs resting on a sloping wall in front of them (2). Animals were habituated to the chamber twice, before two baseline readings were taken (average is presented as baseline). Three consecutive readings were acquired over two seconds each for every time point. Values are presented as mean of right hind paw / mean of left hind paw over the three trials. In female mice, static weight-bearing measurements were only started one week before fracture surgery (week 3 after OVX).

### Naturalistic behaviours

To measure naturalistic behaviours, an automated behaviour recording system (Laboras 2.6.2, Metris, Netherlands) was used. The animals were singly housed in home cages on recording platforms with food and water *ad libitum* and their behaviour recorded over the 12-hour night phase. The frequencies of eating, grooming and total behaviours were measured as well as the duration of locomotion and climbing.

1- Chaplan SR, Bach FW, Pogrel JW, Chung JM, Yaksh TL (1994) Quantitative assesment of tactile allodynia in the rat paw. J Neurosci Methods 53: 55-63.

2- Bove SE, Calcaterra SL, Brooker RM, Huber CM, Guzman RE, Juneau PL, et al. (2003) Weight bearing as a measure of disease progression and efficacy of anti-inflammatory compounds in a model of monosodium iodoacetate-induced osteoarthritis. Osteoarthritis and Cartilage 11: 821-30.
